# Maraviroc-Mediated Lung Protection following Trauma-Hemorrhagic Shock

**DOI:** 10.1155/2016/5302069

**Published:** 2016-07-31

**Authors:** Fu-Chao Liu, Chih-Wen Zheng, Huang-Ping Yu

**Affiliations:** ^1^Department of Anesthesiology, Chang Gung Memorial Hospital, Taoyuan 33305, Taiwan; ^2^College of Medicine, Chang Gung University, Taoyuan 33302, Taiwan

## Abstract

*Objectives.* The peroxisome proliferator-activated receptor gamma (PPAR*γ*) pathway exerts anti-inflammatory effects in response to injury. Maraviroc has been shown to have potent anti-inflammatory effects. The aim of this study was to investigate whether PPAR*γ* plays an important role in maraviroc-mediated lung protection following trauma-hemorrhage.* Methods.* Male Sprague-Dawley rats underwent trauma-hemorrhage (mean blood pressure maintained at approximately 35–40 mmHg for 90 minutes), followed by fluid resuscitation. During resuscitation, a single dose of maraviroc (3 mg/kg, intravenously) with and without a PPAR*γ* inhibitor GW9662 (1 mg/kg, intravenously), GW9662, or vehicle was administered. Lung water content, tissue histology, and other various parameters were measured (*n* = 8 rats/group) 24 hours after resuscitation. One-way ANOVA and Tukey's testing were used for statistical analysis.* Results.* Trauma-hemorrhage significantly increased lung water content, myeloperoxidase activity, intercellular adhesion molecule-1, interleukin-6, and interleukin-1*β* levels. These parameters significantly improved in the maraviroc-treated rats subjected to trauma-hemorrhage. Maraviroc treatment also decreased lung tissue damage as compared to the vehicle-treated trauma-hemorrhaged rats. Coadministration of GW9662 with maraviroc abolished the maraviroc-induced beneficial effects on these parameters and lung injury.* Conclusion.* These results suggest that PPAR*γ* might play a key role in maraviroc-mediated lung protection following trauma-hemorrhage.

## 1. Introduction

The lungs are a vital hollow organ with a large network of vessels and alveoli [1]. Trauma-hemorrhagic shock (THS) can cause vessels to collapse and lead to the production of massive amounts of proinflammatory mediators, resulting in immune cell infiltration and activation, pulmonary tissue damage, changes in capillary permeability, and, subsequently, irreversible lung dysfunction [2, 3]. Severity of lung injury is associated with poor outcome following trauma-hemorrhage [2, 4].

Cysteine-cysteine chemokine receptor type 5 (CCR5) is a common chemokine ligand receptor involved in inflammation that is expressed in different cells [5]. A previous study showed that CCR5 plays a critical role in intestinal ischemia/reperfusion (I/R) injury [6] and endotoxin-induced lung injury [7]. However, the effects of a CCR5 antagonist in THS remain unknown. Peroxisome proliferator-activated receptor gamma (PPAR*γ*) possesses anti-inflammatory effects, including decreasing chemokines/cytokines levels, activation of reactive oxygen species, and expression of adhesion molecules [8]. Activation of PPAR*γ* has been shown to decrease lipopolysaccharide-induced acute lung injury [9], to ameliorate adult respiratory distress syndrome and chronic obstructive pulmonary disease [10], and to have hepatoprotective effects following trauma-hemorrhage [11]. It is inferred that PPAR*γ* plays a role in attenuating organ injury following THS. Maraviroc is a potent CCR5 antagonist that may attenuate organ injury following an allograft [12]. To evaluate the effects of maraviroc and clarify the role of PPAR*γ* in lung injury following THS, we studied maraviroc alone and in combination with the PPAR*γ* antagonist GW9662 in a THS rat model, by evaluating a variety of pulmonary injury parameters including myeloperoxidase (MPO) activity and levels of intercellular adhesion molecule-1 (ICAM-1), interleukin-6 (IL-6), and IL-1*β*.

## 2. Material and Methods

### 2.1. Animals

Adult male Sprague-Dawley strain rats obtained from the National Science Council Experimental Animal Center of Taiwan were used in this study. All protocols and procedures were approved by the Institutional Animal Care and Use Committee of Chang Gung Memorial Hospital. All animal experiments were performed according to the guidelines of the* Animal Welfare Act* and* Guide for the Care and Use of Laboratory Animals* from the National Institutes of Health.

### 2.2. Rat Trauma-Hemorrhagic Shock Model

A rat model of THS without heparinization was used in this study [13]. Forty-eight male Sprague-Dawley rats (275–325 g) were randomly divided into 6 separate groups (*n* = 8/group). All animals were placed in individual cages in an air-conditioned animal facility (controlled humidity 70–75%, temperature 24–25°C) with a 12-hour light-dark cycle (lights on from 06:00 to 18:00). The animals were allowed to acclimate to the environment for at least one week. Prior to conducting the experiment, all rats were fasted overnight but allowed free access to water. The trauma-hemorrhage and resuscitation procedures were performed as described previously [13]. Experimental rats were anesthetized by 0.9 to 1.1% isoflurane inhalation (Attane, Minrad Inc., Bethlehem, PA) at a flow rate of 1 L/min. Tissue trauma was induced by a 5 cm midline abdominal laparotomy, and the abdominal wound was then closed in layers. Polyethylene catheters (PE-50; Becton Dickinson & Co., Sparks, MD) were inserted in bilateral femoral arteries and the right femoral vein* via* inguinal incision wounds (approximately 0.5 cm in length) followed by closing of the bilateral inguinal incision sites. All wounds were infiltrated with 1% lidocaine (Elkins-Sinn Inc., Cherry Hill, NJ) during the surgical procedure to decrease postoperative pain. The rats were then allowed to awaken and bled rapidly within 10 minutes while maintaining mean arterial pressure of 35 to 40 mmHg. This hypotension level was held until the animals could no longer maintain mean arterial pressure of 40 mmHg unless additional fluid (Ringer's lactate) was administered. This timepoint was defined as the maximal bleed-out, and the amount of withdrawn blood was noted. After maximal bleed-out, mean arterial pressure was maintained between 35 and 40 mmHg until 40% of the maximal bleed-out volume was returned in the form of Ringer's lactate solution (approximately 90 minutes after initial bleeding). The rats were then resuscitated with Ringer's lactate (approximately four times the volume of the shed blood over 60 minutes). Thirty minutes before the end of the resuscitation period, the rats received intravenous (IV) maraviroc (3 mg/kg), maraviroc plus GW9662 (1 mg/kg, IV) at the beginning of resuscitation, GW9662 alone, or an equivalent volume of the vehicle (DMSO: about 0.2 mL). After resuscitation, the catheters were removed, the blood vessels were ligated, and the skin incisions were closed. All sham-operated animals underwent the same procedures; vehicle or maraviroc was also administered to sham-operated rats after catheters were placed, but neither hemorrhage nor resuscitation was performed. The animals were humanely sacrificed at 24 hours with an overdose of sodium pentobarbital (150 mg/kg, Nembutal, Abbott Laboratories) after the end of resuscitation or sham operation. No antibiotics or other analgesics, other than xylocaine, were used in any of the procedures. All animals were observed closely and did not present signs of pain in the experimental period.

### 2.3. Water Content Assay of the Lungs

Twenty-four hours following the THS or sham operation, the rats were anesthetized with isoflurane and then sacrificed. The chest wall was opened, and the left side of the lungs was obtained after clamping the helium. In a separate cohort, the left upper lobe of the lungs was weighed and subsequently dried for 24 hours at 80°C. The water content of the lung tissue was calculated using the wet/dry weight ratio [13].

### 2.4. Measurement of MPO Activity in the Lungs

To assess the pulmonary MPO activity, the whole lungs were homogenized and activity was determined as previously described [13]. Frozen tissue samples (100 mg wet weight) were thawed and suspended in phosphate buffer (1 mL, 50 mM, pH 6.0) containing 0.5% hexadecyltrimethylammonium bromide (Sigma, St. Louis, MO). The samples were sonicated on ice and centrifuged at 2,000 ×g for 15 minutes at 4°C, and the supernatants were stored at −80°C (Bio-Rad DC Protein Assay, Bio-Rad, Hercules, CA). MPO assays were carried out in a 96-well plate with the addition of phosphate buffer (290 *μ*L, 50 mM), peroxidase substrate solution (3 *μ*L, containing 20 mg/mL* o*-dianisidine hydrochloride), and H_2_O_2_ (3 *μ*L, 20 mM) followed by the addition of supernatant sample (10 *μ*L) to each well to start the reaction. Standard control MPO was used in parallel to determine the relative MPO activity in the sample. The reaction was stopped by adding sodium azide (3 *μ*L, 30%). Absorbance was measured at 460 nm, and the MPO activity was determined by using the standard curve obtained from the control MPO.

### 2.5. Histological Examination of the Lungs

To examine lung histology, three pieces of the left lung were fixed in 10% formalin in phosphate-buffered saline for 24 hours and were sent to the histology laboratory for further processing. The sections were briefly embedded in paraffin, cut (4-5 *μ*m thickness), and mounted onto glass slides. Lung sections were stained with hematoxylin-eosin and observed under a microscope (Nikon Eclipse TS100) at 200x magnification. Changes in lung morphology were recorded and photographed (SPOT, RTcolor, Diagnostic Instrument, Inc., Iowa City, Iowa) using a microscope-attached camera.

### 2.6. Measurement of ICAM-1, IL-6, and IL-1*β* Levels in the Lungs

Lung ICAM-1, IL-6, and IL-1*β* levels were determined by commercial ELISA (R&D, Minneapolis, MN) according to the manufacturer's instructions as described previously [13]. The lung tissues were briefly homogenized in PBS (weight/volume: 1/10, pH 7.4) containing protease inhibitors (Complete Protease Inhibitor Cocktail, Boehringer Mannheim, Germany). The homogenates were centrifuged (2000 ×g, 20 minutes, 4°C), and the supernatant was assayed for ICAM-1, IL-6, and IL-1*β* levels. An aliquot of the supernatant was used to determine protein concentration with the Bio-Rad DC Protein Assay (Bio-Rad, Hercules, CA).

### 2.7. Statistical Analysis

The InStat 3.0 biostatistics program (GraphPad Software Inc., San Diego, CA) was used for statistical analysis. Results are presented as the mean ± the standard error of the mean (SEM). The data were analyzed using one-way analysis of variance (ANOVA) and Tukey's range test. A *p* value of ≤ 0.05 was considered statistically significant.

## 3. Results

### 3.1. Alteration of Water Content in the Lungs

**Figure 1:**
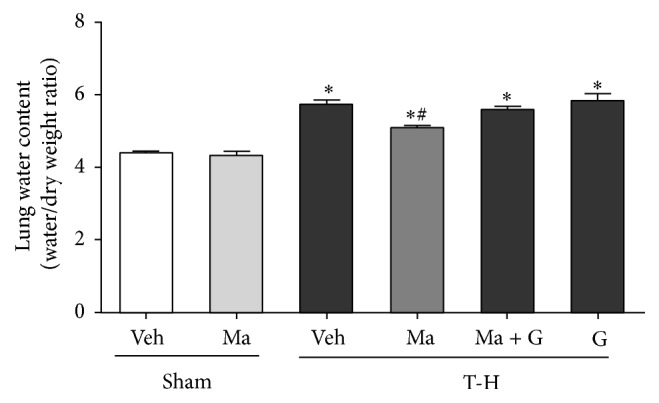
Lung water content (wet to dry) in rats at 24 hours after sham operation (Sham) or trauma-hemorrhage and resuscitation (T-H). Animals were treated with either vehicle (Veh), maraviroc (Ma), maraviroc in combination with GW9662 (Ma + G), or GW9662 (G). Data are shown as mean ± SEM of 8 rats in each group. ^*∗*^ *p* < 0.05 compared to Sham; ^#^ *p* < 0.05 compared to T-H + Veh, T-H + Ma + G, and T-H + G.

As shown in Figure 1, no significant difference in water content (wet/dry weight ratio) was observed between vehicle- and maraviroc-treated sham groups. At 24 hours after trauma-hemorrhage, there were significant increases in lung water content. Administration of maraviroc (3 mg/kg body weight) significantly reduced the increase in the wet/dry weight ratio compared to the vehicle control. To determine whether the beneficial effects of maraviroc in attenuating lung injury after THS were mediated* via* PPAR*γ*-mediated activity, a group of maraviroc-treated THS rats were given the PPAR*γ* antagonist GW9662. The results indicated that administration of GW9662 prevented the maraviroc-induced decrease of water content in the lungs.

### 3.2. Alteration of MPO Activity in the Lungs

**Figure 2:**
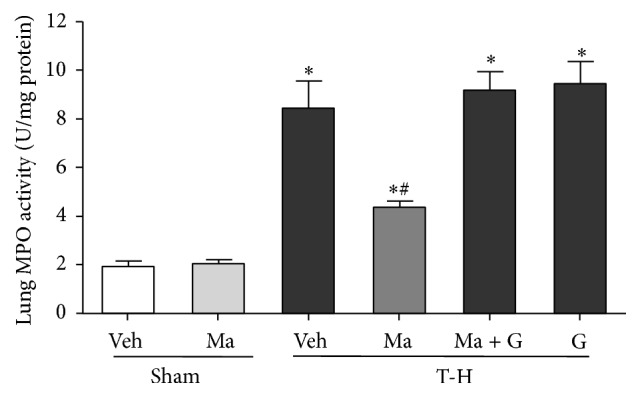
Effect of maraviroc treatment on lung MPO activity in rats at 24 hours after sham operation (Sham) or trauma-hemorrhage and resuscitation (T-H). Animals were treated with either vehicle (Veh), maraviroc (Ma), maraviroc in combination with GW9662 (Ma + G), or GW9662 (G). Data are shown as mean ± SEM of 8 rats in each group. ^*∗*^ *p* < 0.05 compared to Sham; ^#^ *p* < 0.05 compared to T-H + Veh, T-H + Ma + G, and T-H + G.

Lung MPO activity in sham or trauma-hemorrhaged animals, with and without maraviroc treatment, is shown in Figure 2. In sham-operated rats, maraviroc did not alter hepatic MPO activity. THS resulted in a significant increase in lung MPO activity in vehicle-treated animals, while maraviroc treatment attenuated the increase in lung MPO activity. Furthermore, administration of GW9662 prevented the maraviroc-mediated attenuation of lung MPO activity after THS.

### 3.3. Alteration of ICAM-1 Expression in the Lungs

**Figure 3:**
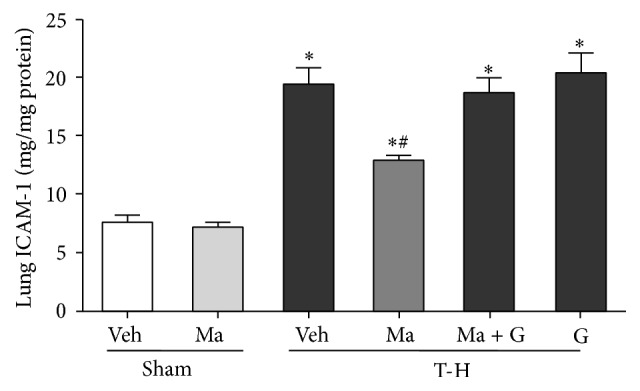
ICAM-1 activities in the lungs of rats after sham operation (Sham) or trauma-hemorrhage and resuscitation (T-H). Animals were treated with either vehicle (Veh), maraviroc (Ma), maraviroc in combination with GW9662 (Ma + G), or GW9662 (G). Data are shown as mean ± SEM of 8 rats in each group. ^*∗*^ *p* < 0.05 compared to Sham; ^#^ *p* < 0.05 compared to T-H + Veh, T-H + Ma + G, and T-H + G.

THS significantly increased ICAM-1 concentration in the lungs (Figure 3). Treatment with maraviroc attenuated the THS-induced increase in ICAM-1 concentration. Coadministration of the PPAR*γ* antagonist GW9662 with maraviroc prevented the maraviroc-induced reduction in ICAM-1 concentration.

### 3.4. Alteration of IL-6 and IL-1*β* Levels in the Lungs

**Figure 4:**
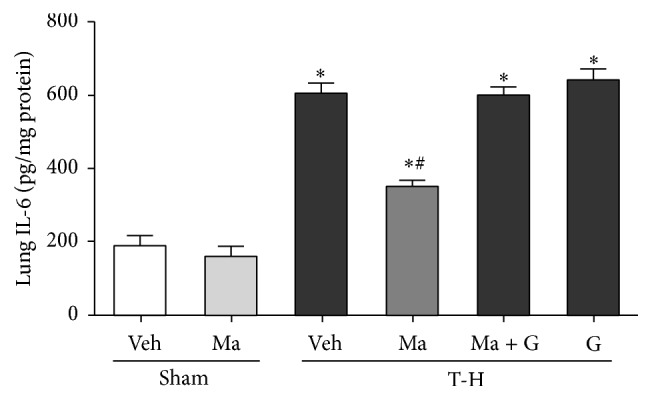
IL-6 levels in the lungs of rats after sham operation (Sham) or trauma-hemorrhage and resuscitation (T-H). Animals were treated with either vehicle (Veh), maraviroc (Ma), maraviroc in combination with GW9662 (Ma + G), or GW9662 (G). Data are shown as mean ± SEM of 8 rats in each group. ^*∗*^ *p* < 0.05 compared to Sham; ^#^ *p* < 0.05 compared to T-H + Veh, T-H + Ma + G, and T-H + G.

**Figure 5:**
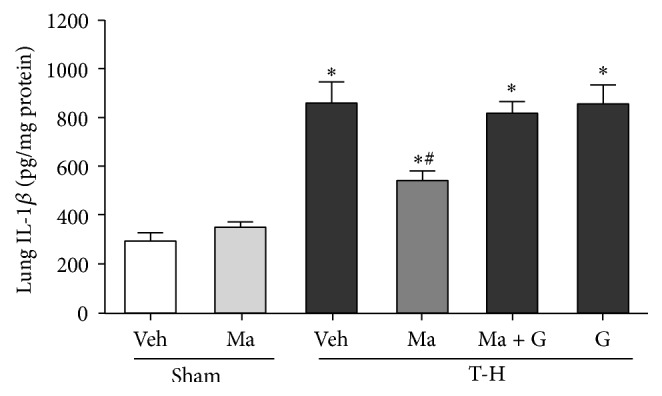
IL-1*β* expression in the lungs of rats after sham operation (Sham) or trauma-hemorrhage and resuscitation (T-H). Animals were treated with either vehicle (Veh), maraviroc (Ma), maraviroc in combination with GW9662 (Ma + G), or GW9662 (G). Data are shown as mean ± SEM of 8 rats in each group. ^*∗*^ *p* < 0.05 compared to Sham; ^#^ *p* < 0.05 compared to T-H + Veh.

There was no significant difference in hepatic IL-6 and IL-1*β* levels between the vehicle- and maraviroc-treated sham groups (Figures 4 and 5). THS significantly increased lung IL-6 and IL-1*β* in vehicle-treated rats as compared to the sham animals. The increase in IL-6 and IL-1*β* levels was attenuated by maraviroc treatment, and the maraviroc-mediated reduction in IL-6 and IL-1*β* levels was abolished by coadministration of GW9662.

### 3.5. Histological Analysis of the Lungs

**Figure 6:**
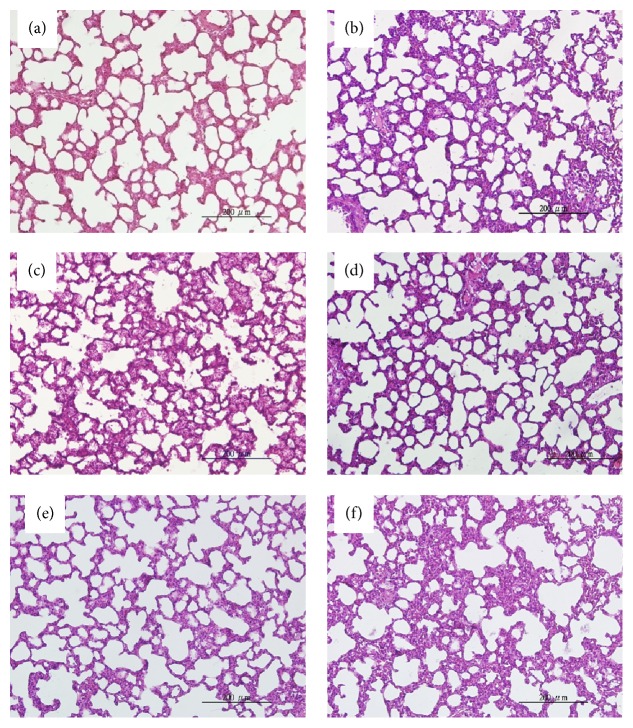
Representative photomicrographs of lungs of sham animals receiving vehicle (a), sham animals receiving maraviroc (b), trauma-hemorrhage animals receiving vehicle (c), trauma-hemorrhage animals receiving maraviroc (d), trauma-hemorrhage animals receiving maraviroc and GW9662 (e), and trauma-hemorrhage animals receiving GW9662 (f). Animals were sacrificed 24 hours after sham operation or trauma-hemorrhage with resuscitation. Lungs were removed and processed as described in Section 2. Lung sections were stained with hematoxylin-eosin, examined at an original magnification of ×200, and photographed.

Representative photomicrographs of the lungs were taken for sham animals treated with vehicle (Figure 6(a)), sham animals treated with maraviroc (Figure 6(b)), trauma-hemorrhage animals treated with vehicle (Figure 6(c)), trauma-hemorrhage animals treated with maraviroc (Figure 6(d)), trauma-hemorrhage animals receiving coadministered maraviroc and GW9662 (Figure 6(e)), and trauma-hemorrhage animals receiving GW9662 alone (Figure 6(f)). Similar results were obtained from four or more animals in each group. These results suggest that maraviroc ameliorated THS-induced lung damage.

## 4. Discussion

In the present study, we investigated whether PPAR*γ* plays an important role in maraviroc-mediated lung protection following THS in male rats. Maraviroc, a CCR5 antagonist, has been used for evaluating the effects of CCR5 inhibition in lung injury following THS. We demonstrate that treatment with maraviroc (3 mg/kg) attenuated THS-induced lung injury by employing a dose obtained from our previous study [11]. Pulmonary MPO activity, ICAM-1, IL-6, and IL-1*β* levels significantly increased 24 hours after THS. However, the increases in these parameters were attenuated by treatment with maraviroc during resuscitation. Furthermore, our results indicated that administration of maraviroc combined with the PPAR*γ* antagonist GW9662 abolished the maraviroc-produced pulmonary protective effect in THS rats. These findings collectively suggest that a PPAR*γ*-related pathway may play a key role in maraviroc-mediated lung protection following THS.

Traumatic injuries and severe blood loss can produce respiratory impairment and development of delayed pulmonary dysfunction [3]. Previous studies have shown that lung injury is associated with neutrophil infiltration and accumulation following THS [13, 14]. Massive neutrophil infiltration and activation can induce a series of immune and intercellular reactions, which are accompanied by increased cytokine release and adhesion molecule expression [15]. MPO is an important indicator of neutrophil infiltration and is correlated with adhesion molecule expression and the severity of tissue injury following THS [13, 16]. Our results indicate that the increase in MPO activity and ICAM-1 levels was attenuated in maraviroc-treated rats following THS.

IL-6 is an important component in inflammatory and immune response that is correlated with* neutrophil* recruitment to infected or injured tissue [17]. Previous studies have shown that pulmonary neutrophil infiltration decreases in IL-6-deficient mice compared to that in wild-type mice in hemorrhagic shock [18]. IL-1*β* is responsible for mediating several immune responses and may contribute to the development of inflammation during organ injury [19, 20]. IL-1*β* may stimulate alveolar macrophage IL-8 expression and accumulate neutrophils into the lungs [21] and increase the expression of human pulmonary epithelial cell adhesion molecule ICAM-1 as part of the inflammatory response [22]. In the present study of maraviroc-treated rats following THS, the reduced accumulation of lung neutrophils was accompanied by a decrease of cytokine and adhesion molecule production, including IL-1*β*, IL-6, and ICAM-1.

Maraviroc is a CCR5 receptor antagonist, which is often used clinically in the treatment of HIV infections [23]. Previous studies have shown that maraviroc can inhibit lipopolysaccharide-induced proinflammatory cytokine expression in human adipocytes [24], formyl-methionyl-leucyl-phenylalanine-induced chemotactic activity of innate immune cells [25], and vascular cell adhesion molecule-1 levels in HIV-infected patients [26]. Martin-Blondel et al. [27] have shown that maraviroc offers neuroprotective benefits in neuroinflammatory diseases. Our previous report also demonstrated that maraviroc had a hepatoprotective effect following trauma-hemorrhage [11]. Recent studies have further shown that maraviroc treatment can reduce pulmonary intravascular, interstitial, and alveolar neutrophil infiltration in endotoxin-induced lung injury [7] and that it can protect against complement component 5a-induced acute lung injury [28].

PPAR*γ*, a member of the nuclear hormone receptor superfamily, is expressed in a variety of different cells including monocytes, macrophages, fat cells, and endothelial cells [29]. Previous evidence has shown that PPAR*γ* affects proinflammatory cytokine production and neutrophil migration in response to injury [11, 30]. Additionally, PPAR*γ* plays an important role in shock-induced lung injuries [31]. Activation of PPAR*γ* can decrease endotoxemic-induced [32], lipopolysaccharide-induced [9], and hemorrhage-induced [31] acute pulmonary inflammation and injury through inhibition of neutrophil accumulation, ICAM-1 expression, and proinflammatory cytokine (IL-6, IL-1*β*) production [33]. Previous evidence has shown that the PPAR*γ* antagonist GW9662 can decrease cell survival and increase organ damage following different types of injuries [34, 35]. Our previous study also showed that GW9662 could decrease maraviroc's hepatoprotective effect after trauma-hemorrhage in a rat model [14].

In this study, the protective effects of maraviroc after THS were abolished by coadministration of GW9662. This result suggests that the beneficial effects of maraviroc on pulmonary function are, in part, mediated by a PPAR*γ*-dependent pathway. Furthermore, pulmonary neutrophil activity and lung ICAM-1, IL-6, and IL-1*β* levels were attenuated by maraviroc treatment following THS, whereas maraviroc coadministered with GW9662 reversed these effects. This finding indicates that a PPAR*γ*-dependent pathway also plays a key role in maraviroc-mediated attenuation of the THS-induced pulmonary inflammation and injury.

The THS is usually an unexpected accident. Earlier management and treatment may improve the outcome of patient recovery. Injured patients are sent to hospitals to receive medical treatment usually during the resuscitation period following THS. In our study, we utilized a well-established rat THS model first described by Singh et al. in 1991 [36]. Though other animal models of THS have also been previously reported (dog [37], pig [38], and mice [39]), these models have significant differences and limitations compared to the rat THS model in a number of important respects, including hypotension level, shock period, bleed-out volume, resuscitation fluid variation, and catheter insertion position or bleed-out site [40, 41]. In this hemorrhagic shock and resuscitation rat model, the rats were allowed to rapidly bleed out and hypotension between 35 and 40 mmHg was maintained; Ringer's lactate fluid was used for resuscitation. The protective drug (maraviroc) was administered at the middle of resuscitation. The hemorrhagic shock rat model might be reasonable for clinical condition.

We previously demonstrated that treatment with resveratrol (30 mg/kg, IV) and sirtinol (1 mg/kg, IV) could attenuate lung injury following THS through reduction of expression of proinflammatory mediators [13, 42]. A recent study showed that osthol (a natural coumarin compound, 3 mg/kg, IV) could decrease pulmonary MPO activity and lung injury through suppression of PDE4 expression under the same hemorrhagic shock conditions [43]. In this study, maraviroc (3 mg/kg, IV) treatment decreased lung injury through a PPAR*γ*-dependent pathway in the same hemorrhagic shock rat model. The effective dosage of maraviroc in this model was similar or identical to sirtinol and osthol but significantly lower than resveratrol. However, the drug's efficacy and toxicity remain to be determined. In our study, administration of maraviroc decreased trauma-hemorrhage-induced lung injury and cytokines production in rats. However, the effect of maraviroc on the survival of the animals has not been investigated and needs to be determined in further study.

Briefly, treatment with maraviroc decreased the pulmonary MPO, ICAM-1, IL-6, and IL-1*β* expressions and attenuated lung injury after THS. The beneficial effects of maraviroc were abolished by coadministration of PPAR*γ* antagonist GW9662. These results suggest that the molecular mechanism of maraviroc in lung protection is through the PPAR*γ*-mediated attenuation of neutrophil accumulation, adhesion molecule expression, and cytokine overproduction after THS.

In conclusion, our results indicate that maraviroc administration following THS attenuates production of proinflammatory mediators and cellular adhesion molecules through a PPAR*γ*-related pathway. Our findings suggest that maraviroc may be a novel adjunct for improving lung function under adverse circulatory conditions.
